# Modeling Beta-Traces for Beta-Barrels from Cryo-EM Density Maps

**DOI:** 10.1155/2017/1793213

**Published:** 2017-01-10

**Authors:** Dong Si, Jing He

**Affiliations:** ^1^Division of Computing and Software Systems, University of Washington Bothell, Bothell, WA 98011, USA; ^2^Department of Computer Science, Old Dominion University, Norfolk, VA 23529, USA

## Abstract

Cryo-electron microscopy (cryo-EM) has produced density maps of various resolutions. Although *α*-helices can be detected from density maps at 5–8 Å resolutions, *β*-strands are challenging to detect at such density maps due to close-spacing of *β*-strands. The variety of shapes of *β*-sheets adds the complexity of *β*-strands detection from density maps. We propose a new approach to model traces of *β*-strands for *β*-barrel density regions that are extracted from cryo-EM density maps. In the test containing eight *β*-barrels extracted from experimental cryo-EM density maps at 5.5 Å–8.25 Å resolution,* StrandRoller* detected about 74.26% of the amino acids in the *β*-strands with an overall 2.05 Å 2-way distance between the detected *β*-traces and the observed ones, if the best of the fifteen detection cases is considered.

## 1. Introduction

**Figure 1:**
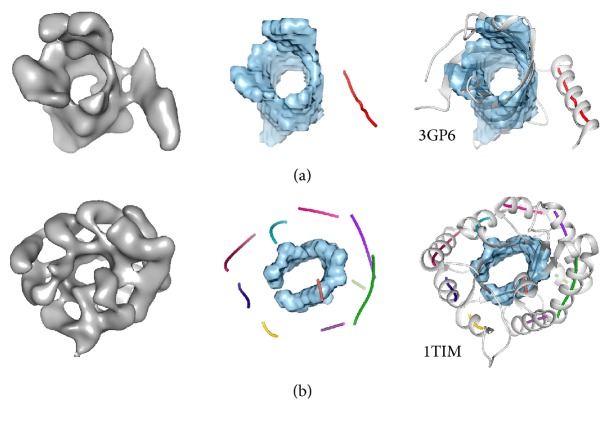
Secondary structure detection from density maps. Left: A density map (gray) was simulated at 10 Å resolution using the atomic structure of protein 3GP6 (PDB ID) in (a), 1TIM (PDB ID) in (b), and EMAN software [38]. Middle: helices (colored lines) and *β*-sheets (blue voxels) are detected from the density maps using* SSETracer* [24]. Right: The detected *α*-helixes and *β*-sheets are superimposed with the atomic structure in (a) and (b), respectively. Each atomic structure contains a *β*-barrel.  Figure 1(a) is reproduced with permission from IEEE [39].

Cryo-electron microscopy (cryo-EM) has become a major experimental technique to study structures of large protein complexes [1, 2]. Many large complexes have been resolved to about 3 Å resolution recently [3, 4], at which the position of protein backbone can be distinguished. For cryo-EM maps at lower resolutions, such as 5–8 Å (referred to as medium resolution in the paper), detailed molecular features are not resolved. It is a challenging problem to derive atomic structures from such density maps. Two types of approaches have been proposed. Fitting relies on a suitable atomic structure [5–9] and de novo modeling relies on the match of secondary structures between those in the density map and those in the protein sequence [10–17]. The most characteristic patterns in cryo-EM density maps at medium resolutions come from secondary structures of a protein chain. An *α*-helix often appears as a cylinder or a stick and can be identified using image processing methods [14, 18–22]. A *β*-sheet consists of multiple *β*-stands (Figure 1). A *β*-sheet often appears as a thin layer of density and can be detected computationally [14, 20, 23–25]. The spacing between two neighboring *β*-strands is between 4.5 and 5 Å, and therefore *β*-strands are not resolved at medium resolutions [26, 27].

**Figure 2:**
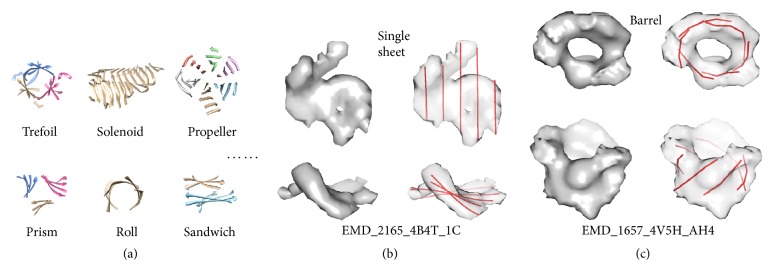
Challenges of *β*-structure modeling from cryo-EM density maps. (a) Various *β*-structures classified in CATH database. (b) An example of an isolated *β*-sheet density map and the *β*-traces predicted using* StrandTwister*. (c) An example of an isolated *β*-barrel density map and the *β*-traces detected using the method proposed in this paper. The top view (upper row) and the side view (bottom row) are shown in (b) and (c).

The position of *β*-strands provides important constraints in backbone modeling of a protein. We previously proposed an approach to predict the location of *β*-strands using* StrandTwister* [28].* StrandTwister* is built on the principle of right-handed twist of *β*-strands that was discovered as early as 1970s [29]. The right-handed twist was measured along the peptide orientation as about 0° to 30° per residue [30], and an estimation of the strand orientation was proposed using the corners of a *β*-sheet [31].* StrandTwister* was able to detect *β*-strands from single *β*-sheets. However, *β*-sheets have a variety of shapes that add complexity for *β*-strands detection. Some *β*-sheets appear as rolls and propellers, and others are *β*-barrels (Figure 2). Currently there is no computational method to detect *β*-strands from a *β*-barrel density map. In this paper, we propose a method to predict *β*-strands by utilizing prior knowledge about *β*-barrels. The proposed method* StrandRoller* is quite different from the method of* StrandTwister*.

A *β*-barrel is a large *β*-sheet in which the first *β*-strand is hydrogen-bonded with the last *β*-strand. *β*-barrels are commonly found in porins and other proteins that span cell membranes [32]. It was noticed by McLachlan in 1979 that the number of strands and their relative stagger completely determines the overall structure of a *β*-barrel [33]. The main structural characteristics of an ideal *β*-barrel have been discussed based on a cylindrical barrel [33–35]. Studies have shown that tilt angle and interstrand distance for all *β*-barrel structures vary within a fairly small range [35–37]. Our method,* StrandRoller*, is designed to utilize such characteristics of tilt angles and interstrand distance.

**Figure 3:**
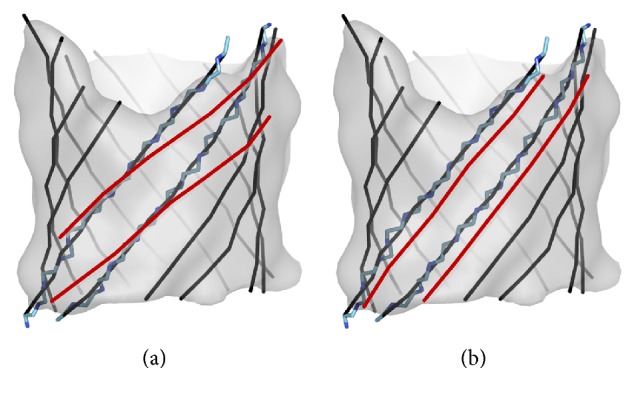
The problem of *β*-barrel modeling from medium resolution density maps. A set of *β*-traces is shown in black lines at the front and gray lines at the back. Two possible sets of *β*-traces (represented by two black lines and two red lines) may have different orientations shown in (a) or locations/shifts shown in (b). The atomic structure of the two *β*-strands is superimposed on the two representative *β*-traces in (a) and (b). Figure 3 is reproduced with permission from IEEE [39].

A helix identified from the medium resolution cryo-EM map is often represented as a line (colored line in Figure 1), referred to as an *α*-trace that corresponds to the central axis of a helix. We define a *β*-trace as the central line along a *β*-strand. In particular, the observed *β*-trace is the line interpolating all geometrical centers of three consecutive C*α* atoms on a *β*-strand plus two C*α* atoms at the ends of the *β*-strand (black line in Figure 3). An observed *β*-trace represents the line along the atomic structure of a *β*-strand (Figure 3). Given the *β*-barrel density voxels, the problem of *β*-strands detection is to find the orientation (Figure 3(a)) and location (Figure 3(b)) of *β*-traces from the three-dimensional cryo-EM map.

Preliminary result of* StrandRoller* was shown in [39]. Details of the method and more thorough tests on different sizes of *β*-barrels are shown in this paper. In addition, we tested our method on eight pieces of experimental cryo-EM data that were downloaded from EM DataBank. The result suggests that* StrandRoller* can be used for the prediction of *β*-traces from medium resolution cryo-EM density maps of *β*-barrel when the rough barrel region is segmented.

## 2. Method

**Figure 4:**
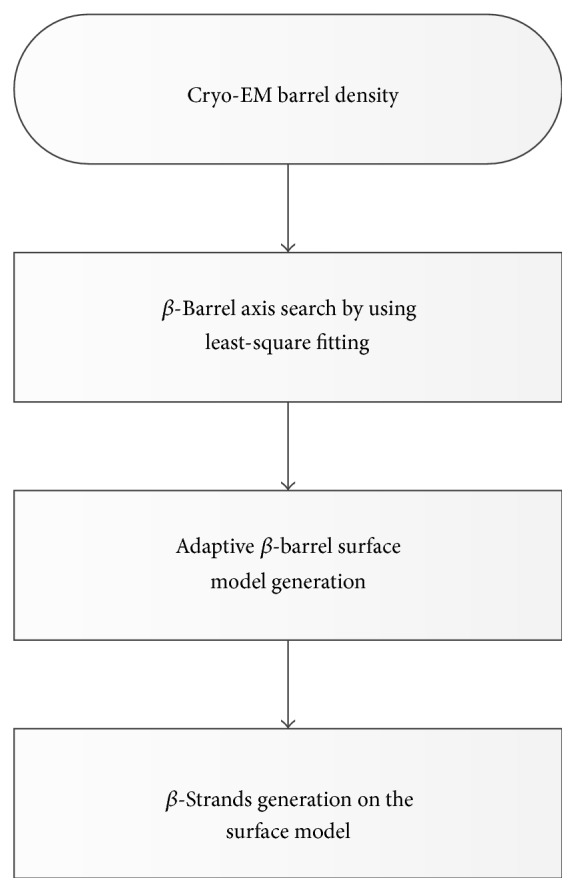
The flow chart of* StrandRoller*.

*β*-Barrels have a characteristic shape. Atomic structures of *β*-barrels have been modeled as hyperboloid surfaces [40–42] or catenoid surfaces [43]. Although atomic structures of *β*-barrels are different from *β*-barrel density maps, many small *β*-barrels visually appear as cylinders with nonuniform ends. Although various models can be used to approximate the major area of a *β*-barrel, *β*-barrel density maps often deviate from the mathematical models at certain regions. In order to create a surface well representing the density map, we used a simple elliptical model initially and adjusted the model at those regions that do not fit. Strand generation was then performed on the adjusted barrel surface model (Figure 4). We assume that the *β*-barrel density has been segmented from the entire cryo-EM density map.

### 2.1. *β*-Barrel Surface Model from Cryo-EM Density Voxels

**Figure 5:**
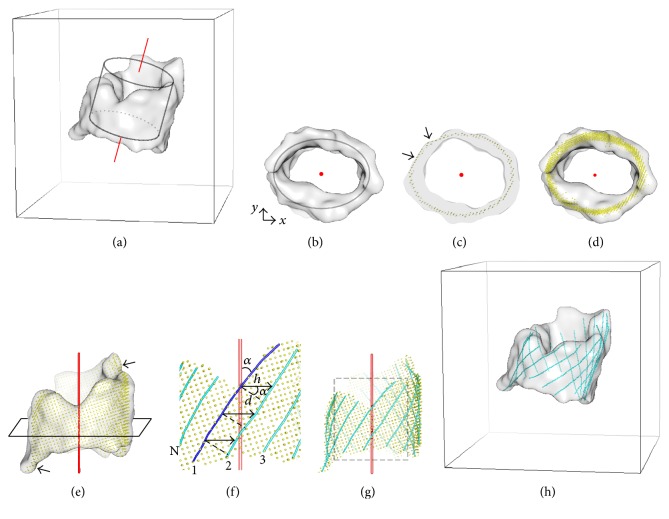
Generation of *β*-barrel model and *β*-traces from a *β*-barrel density map. (a-b) The barrel axis (red) was identified by fitting an elliptical cylinder to the density map (gray). (c) A cross-section of the barrel shows adjustment (arrow) of the ideal elliptical cylinder model at morphed regions. Top view (d) and the side view (e) of the modeled barrel surface (yellow) show the barrel axis (red) and a cross-section of the *β*-barrel. The zoom-in view (f) and the entire-view (g) of the *β*-traces generated recursively on the surface model using tilt angle *α* and interstrand distance *d* of *β*-strands. *β*-Traces are superimposed with the *β*-barrel density map in (h).  Figure 5 is reproduced with permission from IEEE [39].

In order to represent the shape of a *β*-barrel, we reduced the *β*-barrel density map to a surface. Two steps are involved in creating the surface model. The first step involves identification of the axis of the barrel density map (Figure 5(a)). The barrel density map was first translated to global origin (0, 0, 0) based on its geometric center. An elliptical cylinder (1) was then utilized to search for the orientation of central axis. The orientation was selected using exhaustive search and least square fitting to the cylinder. The entire barrel density was then rotated such that the central axis aligns with the *z*-axis (Figure 5(b)).

(1) $$\begin{array}{c} \frac{x^{2}}{a^{2}}+\frac{y^{2}}{b^{2}}=1. \end{array}$$

Instead of a mathematical formula, our *β*-barrel model consists of a thin layer of density voxels that closely represents the morphed barrel shape and outline of barrel at two ends (yellow points in Figure 5(e)). The barrel model was generated using cross-sections from bottom to top of the volume. The density voxels on each cross-section of *Z*-axis (Figure 5(c), gray) appear to be nearby the ideal model of ellipse. The voxels that are closest to the ideal ellipse were selected as the points on barrel model (Figure 5(c), yellow). Note that such a discrete model closely represents the three-dimensional distribution of the voxels. For example, when the fitted ideal elliptical cylinder is outside the density (arrows in Figure 5(c)), the voxels on the density map were used to adjust the barrel model. It appears that the resulting barrel model clearly represents the morphed regions, especially at the two ends of barrel (arrows in Figure 5(e)). We find that it is important to have an accurate barrel surface to model the *β*-traces accurately.

### 2.2. Strand Generation on the Barrel Model

McLachlan noticed in 1979 that the number of strands and their relative stagger completely determines the overall structure of a *β*-barrel [33]. The main structural characteristics of ideal *β*-barrel have been discussed based on a cylindrical barrel [33–35]. Studies have shown that tilt angle *α* (Figure 5(f)) of a *β*-strand can vary between 30° and 60°, as reflected in the known structures of membrane proteins [35–37]. The tilt angle *α* may vary by ±15° for different strands in the same *β*-barrel [37]. However, the interstrand distance *d* remains to be 4.5~5 Å due to hydrogen bonds between two neighboring *β*-strands.* StrandRoller* uses previous knowledge about the tilt angle *α* and the interstrand distance *d* in the modeling of *β*-strands.

An initial *β*-trace (blue in Figure 5(f)) was produced by tilting the barrel axis with  *α* and projecting it onto the barrel surface model. The second *β*-trace was then generated from the previous one by traveling a horizontal distance *h* (2) on the barrel surface (Figure 5(f)).

(2) $$\begin{array}{c} h=\frac{d}{\cos\alpha },\;d=4.8\;Å. \end{array}$$

Given a tilt angle, the entire set of *β*-traces can be built iteratively on the barrel surface (Figure 5(g)) until the last *β*-trace is generated (Figure 5(f)). The tilt angle was sampled every 5° between 35° and 55°, and three translations were sampled at each tilt angle. There are fifteen sets of *β*-traces generated for one barrel volume. Note that the fifteen sets are within a very small range of tilt angle (20°) and translation distance (4.8 Å). The barrel density along with the detected *β*-strands was eventually translated and rotated back to the original position in the map after the detection is done (Figure 5(h)).

## 3. Result

*StrandRoller* was tested using three sets of *β*-barrel density maps: eighteen small simulated maps, fourteen large simulated maps, and eight experimental cryo-EM *β*-barrel maps. The proteins used in the simulated test set were collected from the *β*-barrel transmembrane superfamily of Orientations of Proteins in Membranes (OPM) database [44] with less than 40% sequence similarity. The atomic structures of *β*-barrels were used to generate *β*-barrel density maps at 10 Å resolution using the pdb2mrc function in EMAN [38], with a sampling of 1 Å/pixel. The experimental cryo-EM density maps were downloaded from EMDB (http://www.emdatabank.org/). Since atomic structures are available for such cryo-EM maps, the density region that corresponds to one chain of the protein was first segmented using the atomic structure as an envelope. The *β*-sheet voxels were then manually outlined based on the atomic structure of the *β*-barrel. Such segmented testing maps bare the characteristic of a *β*-sheet and have an outline of a *β*-barrel. The accuracy of *β*-strand detection was evaluated using two parameters as previously implemented [28]: 2-way distance between the set of detected *β*-traces and the set of observed *β*-traces and number of amino acids covered in the detected *β*-trace. The observed *β*-trace is the line interpolating all geometrical centers of three consecutive C*α* atoms on a *β*-strand plus the two C*α* atoms at the ends of *β*-strand, as shown in Figure 3.

In order to estimate how much of a *β*-strand was detected, the percentage of the detected C*α* atoms of an observed *β*-strand was calculated. An amino acid of a *β*-strand is considered detected if the projection distance from its C*α* atom to the corresponding detected *β*-trace is less than 2.5 Å, which is about half *β*-strand spacing. Since the number of detected *β*-strands may be different from the number of observed *β*-strands, one-to-one correspondence needs to be established between subsets of the *β*-traces. For example, if detected set contains five *β*-traces while observed set contains six *β*-traces, five out of the six observed *β*-traces which have the overall smallest 2-way distance with the five detected *β*-traces will be selected for the calculation of 2-way distance. This ensures that the same number of detected *β*-traces (*S*_1_, *S*_2_,…, *S*_*T*_) is compared to the same number of observed *β*-traces (*S*_1_′, *S*_2_′,…, *S*_*T*_′) in which *S*_*k*_ is compared with *S*_*k*_′ for  *k* = 1, …, *T*. The number of misdetected (and/or wrongly detected) *β*-strands can be inferred from the difference between the total number of the observed and that of the detected *β*-traces. The 2-way distance of a *β*-strand  *k*, *D*_*k*_ was calculated for each pair of lines *S*_*k*_ and *S*_*k*_′. The overall 2-way distance *D* reflects how far the two sets of *β*-traces (detected and observed) are from each other.

(3) Dk=∑i=1NDiss′/N+∑j=1MDjs′s/M2

(4) D=∑s=1TDkT.

In formula (3), *N* and *M* are the numbers of points on detected *β*-traces (*S*_*k*_) and observed *β*-traces (*S*_*k*_′), respectively. *i* and *j* are the indices of a point along lines *S*_*k*_ and *S*_*k*_′, respectively. *D*_*i*_^*ss*′^ is the projection distance from point *i* of *S*_*k*_ to *S*_*k*_′. The projection of point *i* is required to be within line *S*_*k*_′. In case it is outside, the distance between *i* and an end of *S*_*k*_′ was used as an approximate distance.

### 3.1. Performance on the Simulated Density Data

The purpose of this test is to investigate if traces of *β*-strands can be modeled from *β*-barrel density maps simulated to 10 Å resolution, at which the separation of *β*-strands is not visible. To discuss the ability of our *β*-trace detection, we use the best of fifteen sampled sets. The best set is the one that is closest to the observed set in terms of 2-way distance.

#### 3.1.1. Small-Medium Barrels

**Figure 6:**
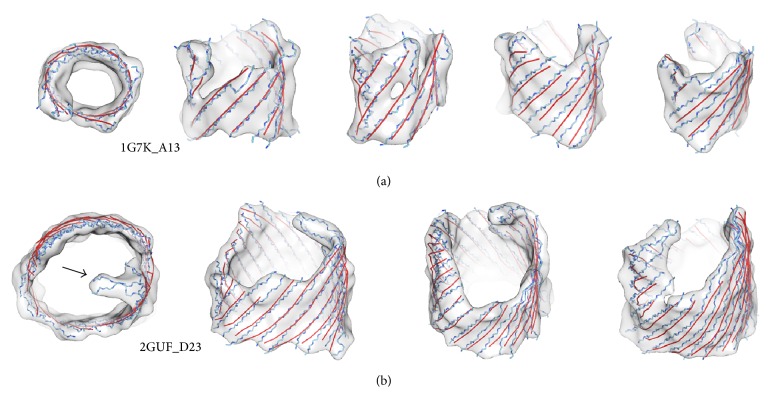
*β*-Strands modeling from the simulated density map at 10 Å. The best of the fifteen sets of modeled *β*-traces (red) are superimposed with the backbone of *β*-strands (blue) and the density maps (gray) for *β*-barrels 1G7K_A13 in (a) and 2GUF_D23 in (b). The top view (left) and four side views are shown in each case.

Small-medium *β*-barrels refer to those with less than 15 *β*-strands in each. The test of eighteen simulated small-medium sized *β*-barrel density maps shows that one of the fifteen sets of *β*-traces aligns very well with the observed set of *β*-traces, with an overall 2-way distance of 1.61 Å for the detected *β*-traces (Table 1). In the case of sheet A13 of PDB structure 1G7K, the detected set of *β*-traces appears to align with the *β*-strands very well (Figure 6(a)). In this case, all the eleven strands were detected with a small 2-way distance of 1.8 Å (Table 1 row 1).

To analyze the sensitivity of the detection, we calculated the percentage of the detected C*α* atoms of an observed *β*-strand. For example, 1TX2_B has all eight *β*-strands detected (Table 1 row 5). It missed three amino acids. For the eight detected *β*-strands, the 2-way distance is only 0.92 Å. Among the eighteen test cases,* StrandRoller* appears to be able to detect 78.26% of the *β*-strands fairly accurately in one of the fifteen sampled sets of *β*-traces (Table 1). Seventeen test cases have the number of *β*-strands detected the same as observed. The number of detected amino acids and 2-way distance are two parameters that have been used previously in accuracy measurement. Length-association method was proposed recently and can be a potentially more sensitive method to evaluate secondary structure detection [45].

#### 3.1.2. Large Barrels

Large barrels in this paper refer to those with more than 15 *β*-strands. Large barrels appear to be more challenging. Some extremely large *β*-barrels, such as the 22-stranded *β*-barrels 2GUF_D23 and 2HDI_D23 (Table 2), were still well detected. The 2-way distance is 2.03 Å in the case of 2GUF_D23 (Table 2 row 1) and 1.93 Å in the case of 2HDI_D23 (Table 2 row 2). *β*-Barrel 2GUF_D23 has twenty-one of twenty-two *β*-strands detected (Table 2 row 1). It missed sixty-six amino acids in which most are at the edge (arrows in Figure 6(b)). For the twenty-one detected *β*-strands, the 2-way distance is 2.03 Å. Among the fourteen test cases of large sized *β*-barrels,* StrandRoller* appears to be able to detect 69.46% of the *β*-strands fairly accurately in one of the fifteen possible sets of *β*-traces, with an overall 2-way distance of 2.12 Å for the detected *β*-traces (Table 2).

It is noticed that the performance of* StrandRoller* is better on small-medium sized barrel than on the large sized barrel. Large *β*-barrels are more likely to adopt flexible shapes. The missing detection appears to be more at the edge of large *β*-barrels, where the *β*-strands tend to be more flexible (arrows in Figure 6(b)). The number of *β*-strands in large *β*-barrels also tends to be hard to detect due to the error accumulated during strand generation step. Since each *β*-trace is deducted from the previous generated one, error could be propagated while traveling around the barrel.

### 3.2. Performance on Experimental Cryo-EM Data

**Figure 7:**
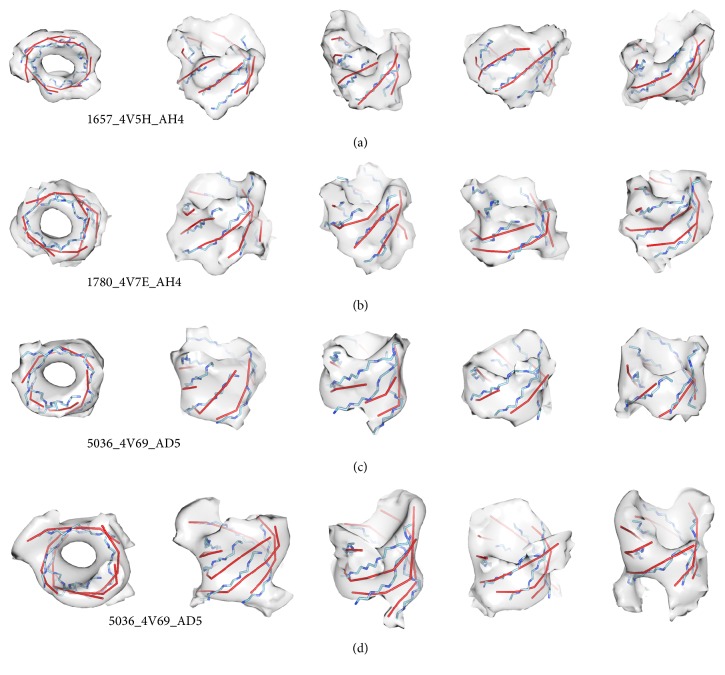
*β*-Traces detected from *β*-barrel density maps obtained from experimentally derived cryo-EM density maps. The best of the fifteen sets of modeled *β*-traces (red) are superimposed with the backbone of the *β*-strands (blue) and the density maps (gray) for *β*-barrel 1657_4V5H_AH4 (EMD_1657, sheet AH4 of protein 4V5H) in (a) and 1780_4V7E_AH4 (EMD_1780, sheet AH4 of protein 4V7E) in (b). The top view (left) and four side views are shown in each case. (c) A conservative segmentation for *β*-barrel 5036_4V69_AD5 (EMD_5036, sheet AD5 of protein 4V69) and its corresponding *β*-strands modeling result. (d) A relaxed segmentation of the same *β*-barrel as in (c) superimposed with the backbone and the detected *β*-traces. *β*-Barrels in (c) and (d) are shown using the same density threshold in similar view points.

*StrandRoller* was tested using eight *β*-barrels obtained from experimental cryo-EM density maps. The eight test cases are small ribosomal proteins in which the first *β*-strand is hydrogen-bonded with the last *β*-strand. Experimental data are often more challenging to analyze due to the noise and missing density.  Figure 7 shows three density regions that were segmented from cryo-EM maps at 5.8 Å, 5.5 Å, and 6.7 Å resolutions, respectively. At these resolutions, *β*-strands are not visible in density maps.* StrandRoller* was able to detect all *β*-strands on the barrels and they align fairly well with the observed *β*-traces. In the case of 70S ribosome EMD_1657 (sheet AH4 in protein 4V5H), the 2-way distance for the five-stranded barrel is 1.94 Å, and it detected 25 of 30 amino acids on the *β*-barrel (Figure 7(a)). In the case of 80S Ribosome EMD_1780 (sheet_AH4 in protein 4V7E), the 2-way distance for the six-stranded barrel is 1.88 Å, and it detected 24 of 28 amino acids on the *β*-barrel (Figure 7(b)). We noticed that the eight *β*-barrels in the cryo-EM maps are all small barrels with less than nine *β*-strands.* StrandRoller* appears to be fairly accurate in detection of *β*-strands from such cryo-EM maps with an overall 2.05 Å 2-way distance and 74.26% of amino acids detected (Table 3).

## 4. Discussion

We previously showed that the accuracy of *β*-strand detection is affected by the accuracy of *β*-sheet detection [28]. This is also true in the context of *β*-barrels. A *β*-barrel is a closed structure, and the number of *β*-strands may be estimated from the diameter of the barrel. Figures 7(c) and 7(d) show the *β*-traces detected from *β*-barrel 5036_4V69_AD5 that were manually extracted from the cryo-EM density map using two different segmentations. The segmentation in Figure 7(c) is more conservative than that in Figure 7(d). The relaxed segmentation of the same barrel includes more density volume at the edge of the barrel. Although the 2-way distance is 1.73 Å in the more conservative segmentation versus 1.83 Å in the other, the main difference in the resulting *β*-traces appears to be the length difference. Both detected the same number of *β*-strands with similar orientation and position. Our result suggests that the number of detected *β*-strands is not sensitive to the density segmentation errors at the two ends of *β*-barrel.

## 5. Conclusion

The position of *β*-strands is critical for modeling atomic structures of proteins. However, it has been a challenging problem to detect *β*-strands when no separation of the *β*-strands is visible from the density maps. The variety of shapes of *β*-sheets adds the complexity of this problem. We previously proposed* StrandTwister* to detect *β*-strands from single *β*-sheet using right-handed twist [28]. We propose a new method to predict *β*-strands from a *β*-barrel density map directly using the characteristic tilt angles of the *β*-barrel. This approach bypasses the need to measure twist angles. Our results show that this approach is feasible. As long as the rough density region of a *β*-barrel is isolated from the entire density map, location of *β*-strands can be modeled. However, current limiting factor is the lack of automatic detection methods of *β*-barrels from a cryo-EM density map. In fact a *β*-barrel has a fundamental shape character in which a hole is surrounded by a *β*-sheet. However, accurate detection of *β*-barrels needs to consider different characters of the hole depending on different sizes of *β*-barrels. We are hopeful that such a detection tool will be available in the near future.

*StrandRoller* does not require the resolution of cryo-EM density map to be higher than 5 Å to resolve the separation of *β*-strands. It applies to the maps with lower resolutions. In the test containing eight experimental cryo-EM *β*-barrel maps between 5.5 Å and 8.25 Å,* StrandRoller *detected about 74.26% of the amino acids in the *β*-strands in one of the fifteen sets of predicted traces. We demonstrate again that it is possible to derive *β*-strands from density maps at medium resolutions. To our knowledge,* StrandRoller* is the first method that attempts to address the problem of *β*-strands detection from medium resolution *β*-barrel maps. Future work includes developing more accurate methods in identification of *β*-traces and generating alternative *β*-traces for further evaluation in modeling.

## Figures and Tables

**Table 1:** Accuracy of medium-small size (less than fifteen strands) beta-barrel modeling from simulated density maps at 10 Å resolution.

PDB ID^a^	#Det./#Obs. Strd^b^	2-w Dist.^c^	#Det./#Obs. AA^d^
1G7K_A13	11/11	1.80	85/124
1QD5_A17	12/12	1.93	99/140
1QJP_A	8/8	1.78	70/107
1RRX_A12	12/11	1.81	86/118
1TX2_B	8/8	0.92	31/34
1UYN_XA12	12/12	1.83	144/189
2ERV_A10	8/8	1.17	74/94
2K0L_A	8/8	1.96	57/79
2LHF_A	8/8	1.44	61/79
2QOM_C	12/12	1.71	153/190
2VDF_AA28	10/10	1.61	144/172
2WJR_AA15	12/12	1.50	114/130
2X9K_AA15	14/14	1.63	151/188
3AEH_A15	12/12	1.85	141/194
3FID_A14	12/12	1.42	131/155
3GP6_A	8/8	1.94	60/88
4E1S_A13	12/12	1.39	118/136
4FQE_A	12/12	1.28	121/134

*Average*		*1.61*	*1840/2351 = 78.26%*
*Standard deviation*		*0.29*	

^a^PDB_Sheet ID.

^b^The number of *β*-traces in the best of the fifteen possible sets/the number of *β*-strands in the *β*-sheet of the PDB structure.

^c^The 2-way distance (in Å) between observed *β*-traces and modeled *β*-traces for the best of the fifteen possible sets.

^d^The number of detected/the total number of amino acids in the *β*-barrel.

**Table 2:** Accuracy of large size beta-barrel (more than fifteen strands) modeling from simulated density maps at 10 Å resolution.

PDB ID^a^	#Det./#Obs. Strd^b^	2-w Dist.^c^	#Det./#Obs. AA^d^
2GUF_D23	21/22	2.03	230/296
2HDI_D23	22/22	1.93	226/296
2J1N_AA18	16/16	1.77	146/181
2MPR_A19	19/19	2.49	134/224
2POR_S117	12/16	2.53	94/178
2QDZ_C17	16/16	1.32	177/198
2YNK_AC20	20/18	2.26	133/194
3CSL_C23	23/22	2.59	171/300
3EMN_A20	20/19	1.83	135/183
3PRN_A17	15/17	1.67	131/162
3SYB_A19	19/18	2.12	155/220
4C00_AE17	17/16	2.37	121/202
4GEY_A18	19/16	2.33	116/189
4K3C_C18	18/16	2.43	126/193

*Average*		*2.12*	*2095/3016 = 69.46%*
*Standard deviation*		*0.38*	

^a^PDB_Sheet ID.

^b^The number of *β*-traces in the best of the fifteen possible sets/the number of *β*-strands in the *β*-sheet of the PDB structure.

^c^The 2-way distance (in Å) between the observed *β*-traces and the modeled *β*-traces for the best of the fifteen possible sets.

^d^The number of detected/the total number of amino acids in the *β*-barrel.

**Table 3:** Accuracy of beta-barrel modeling from cryo-EM experimental maps at medium resolutions.

EMD_PDB ID^a^	Res.^b^	#Det./#Obs. Strd^c^	2-w Dist.^d^	#Det./#Obs. AA^e^
1657_4V5H_AH4	5.8 Å	6/6	1.94	25/30
1780_4V7E_AH4	5.5 Å	5/5	1.88	24/28
1829_4V5H_AH4	5.6 Å	8/6	2.11	25/30
1849_4V6K_AE6	8.25 Å	7/6	2.34	24/40
1849_4V6K_AC6		6/5	1.90	26/31
1849_4V6K_AG2		6/6	2.74	31/52
5030_4V68_AF8	6.4 Å	4/5	1.72	24/32
5036_4V69_AD5	6.7 Å	5/5	1.73	23/29

*Average*			*2.05*	*202/272 = 74.26%*
*Standard deviation*			*0.35*	

^a^EMDB_PDB_Sheet ID.

^b^Resolution of the cryo-EM density map.

^c^The number of *β*-traces detected in the best of the fifteen possible sets/the number of *β*-strands in the *β*-sheet of the PDB structure.

^d^The 2-way distance (in Å) between the observed *β*-traces and the modeled *β*-traces for the best of the fifteen possible sets.

^e^The number of detected/the total number of amino acids in the *β*-barrel.
